# MALAT1 predicts the prognosis of severe community-acquired pneumonia in pediatric patients

**DOI:** 10.1186/s12890-024-03157-9

**Published:** 2024-07-25

**Authors:** Mei Yang, Aili Xuan, Qian Liu, Guoji Zhu

**Affiliations:** 1 Department of Pediatrics, First Affiliated Hospital of Bengbu Medical University, Anhui, China; 2 Department of Pediatrics, Weifang Medical College Affiliated Hospital, Shandong, China; 3 Department of Infectious Diseases, Children’s Hospital, Soochow University, No. 92 Zhongnan Street, Jiangsu, Jiangsu Province 215000 China

**Keywords:** MALAT1, Community-acquired pneumonia, Pediatrics, Survival

## Abstract

**Background:**

To evaluate the role of metastasis-associated lung adenocarcinoma transcript 1 (MALAT1) in the prognosis of severe community-acquired pneumonia (CAP) in children.

**Methods:**

According to the median MALAT1 value of 3.2 at baseline, 93 pediatric patients with severe CAP were divided into low (*n* = 46, median MALAT1 level = 1.9) or high (*n* = 47, median MALAT1 level = 4.5) MALAT1 groups. Another 93 age-, gender-, and body mass index (BMI)-matched healthy individuals were included in the control group using the propensity-score matching (PSM) method. A multivariate Cox proportional hazards model was used to explore the association of MALAT1 level with the 28-day mortality after controlling for potential confounding factors.

**Results:**

The MALAT1 expressions were significantly higher in the patients with severe CAP compared with those in the healthy controls (3.2 vs. 0.9, *P* < 0.01). The receiver operating characteristic (ROC) analysis showed that the area under the curve (AUC) was 0.927 when the cut-off value of MALAT1 was 1.5. Moreover, the MALAT1 expressions were substantially lower in survivals than non-survivals (3.8 vs. 2.6, *P* < 0.01), and the multivariate Cox regression analysis indicated a positive association between MALAT1 levels and mortality risk (HR = 3.32; 95% CI: 1.05–10.47; *P* = 0.04).

**Conclusion:**

MALAT1 might be a promising marker for predicting the prognosis of severe CAP in pediatric patients.

## Background

Pneumonia is the single largest infectious cause of death in children worldwide. Although pneumonia-specific mortality substantially decreased in the past decades, it remains the major cause of death in children outside the neonatal period, accounting for 22% of all deaths in children aged 1 to 5 years [1, 2]. Moreover, the pneumonia survivors commonly suffer from disability, morbidity, and readmission [3]. Therefore, exploring novel and accurate biomarkers is necessary to improve prognosis in pediatric pneumonia patients.

Long non-coding RNAs (lncRNAs) are a group of non-coding RNA transcripts with a length of > 200 nucleotides [4]. LncRNAs regulate gene expression at transcriptional, post-transcriptional, epigenetic, and chromatin levels and activate or constrain the expression of target genes by directly binding to them or by recruiting transcription factors [5]. Moreover, many lncRNAs exhibit cell and/or tissue/tumor-specific expression making them excellent candidates for diagnostic and therapeutic applications [6]. Metastasis-associated lung adenocarcinoma transcript 1 (MALAT1) is a large, infrequently spliced lncRNA that is highly conserved amongst mammals and highly expressed in the nucleus [7]. It could regulate various pathophysiological processes of respiratory diseases, including proliferation, invasion, metastasis, and apoptosis by targeting the expression of multiple miRNAs and activating different signaling pathways [4, 8–12]. Though some data displayed the association of MALAT1 levels with survival in adult patients with pneumonia [13], its prognostic value in children has not been identified.

This study aimed to assess the association of MALAT1 level with the prognosis of severe community-acquired pneumonia (CAP) in children.

## Materials and methods

### Patients

This prospective, observational, cohort study was conducted on consecutive pediatric patients (≤ 18 years) with severe CAP admitted into the First Affiliated Hospital of Bengbu Medical University from January 2021 to December 2022. The diagnosis strictly followed the criteria of clinical management guidelines of the Infectious Diseases Society of America (IDSA) [14]. The diagnosis of severe CAP was made if a child has ≥ 1 major or ≥ 2 minor criteria. Major criteria include (1) invasive mechanical ventilation; (2) fluid refractory shock; (3) acute need for noninvasive positive pressure ventilation; and (4) hypoxemia requiring fraction of inspired oxygen (FiO_2_) greater than the inspired concentration or flow feasible in the general care area. Minor criteria include (1) respiratory rate greater than the WHO classification for age; (2) apnea; (3) increased work of breathing (e.g. retractions, dyspnea, nasal flaring, and grunting); (4) PaO2/FiO2 ratio < 250; (5) multilobar infiltrates; (6) Pediatric Early Warning Score (PWES) > 6; (7) altered mental status; (8) hypotension; (9) presence of effusion; (10) comorbid conditions (e.g., hemoglobin SS disease, immunosuppression, and immunodeficiency); and 11) unexplained metabolic acidosis. All children received routine symptomatic treatment, including vital signs monitoring, nutritional support, oxygen inhalation, and antibiotics. Patients were excluded if they were neonates and preterm infants or had other severe preexisting diseases such as chronic infectious or immune disease, malignancy, allergy, and congenital malformation. Patients who refused to attend the study were also excluded.

After controlling for baseline characteristics including age, sex, and body mass index (BMI) using the propensity-score matching (PSM) method, a cohort of healthy children who received a physical examination at the physical examination center of our hospital but without any symptoms or signs of illness were enrolled as the controls.

### Data Collection and Measurement of MALAT-1 expression

The baseline demographic, laboratory, and clinical data were obtained from medical records. All patients were followed up for 28 days by telephone or outpatient review.

The measurement of MALAT-1 expression was in line with a study conducted by He et al. [15] At baseline, 5 mL peripheral blood was collected from each participant, and then total RNA was elicited using TRIzol reagent (Takara, Shanghai, China) following the manufacturer’s protocol. Next, total RNA was reversely transcribed to complementary DNAs by PrimeScript RT reagent Kit (Takara, Shanghai, China), and qPCR was performed using TB Green Fast qPCR Mix (Takara, Shanghai, China). The relative mRNA expression of MALAT-1 was calculated according to the 2^−ΔΔCt^ formula using GAPDH as the internal reference.

### Statistical analysis

This study enrolled 104 patients. Assuming about 30% of participants would die by the time of the primary analysis (with a two-sided type I error rate of 5%) and a 10% non-response and attrition rate, a power of > 90% will be provided to detect a hazard ratio of 2.0.

The receiver operating characteristic (ROC) curves were plotted to explore the discrimination ability of MALAT1 expressions between severe CAP and healthy controls [16]. Kaplan-Meier curve and multivariate Cox regression were employed to evaluate the association of MALAT1 with the 28-day mortality risk. *P*-value < 0.05 was considered statistically significant.

All statistical analyses were performed with the SPSS statistical software program package (SPSS version 22.0 for Windows, Armonk, NY: IBM Corp.).

## Results

Totally 104 patients were assessed for eligibility, out of which 11 cases were excluded due to the refusal of parents (*n* = 5), loss to follow-up (*n* = 3), and severe preexisting diseases (*n* = 3). Finally, 93 patients were included in the analysis and divided into low (*n* = 46) or high (*n* = 47) MALAT1 groups based on the median value of 3.2. A cohort of 93 age-, gender-, and BMI-matched healthy individuals were enrolled as the controls.

**Fig. 1:**
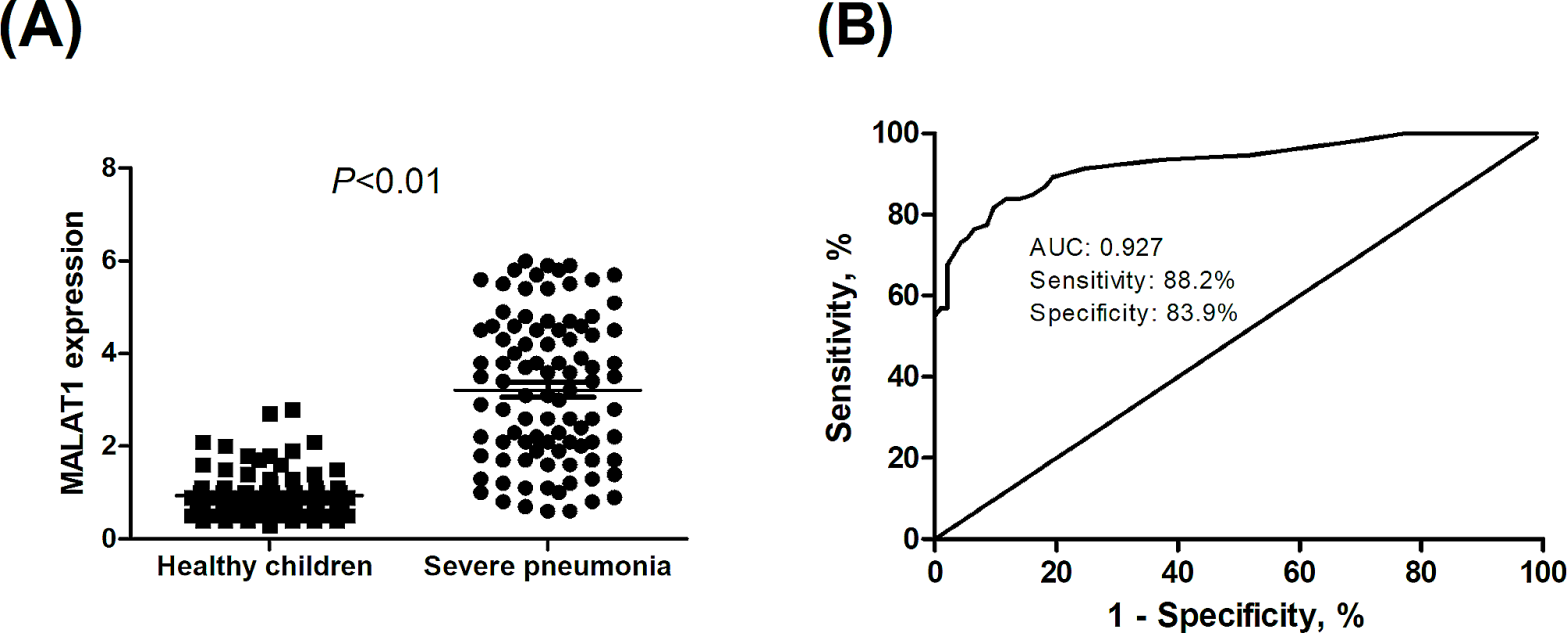
(**A**) Metastasis-associated lung adenocarcinoma transcript 1 (MALAT1) mRNA expression levels in healthy controls and children with severe community-acquired pneumonia; (**B**) the receiver operating characteristic (ROC) curve indicated the predictive significance of MALAT1

As shown in Table 1, there was no significant difference in age, gender, BMI, and white blood cell (WBC) between patients with severe CAP and healthy controls (all *P* > 0.05). Neutrophils, lactate dehydrogenase, lymphocytes, C-reactive protein (CRP), and procalcitonin (PCT) were significantly higher in the patients with severe CAP compared with those in healthy controls (all *P* < 0.01). The MALAT1 expression levels were significantly higher in the patients with severe CAP (3.2 vs. 0.9, *P* < 0.01; Fig. 1A), which propounded that severe CAP might contribute to the obvious abundance of MALAT1 expression. The ROC result showed the predictive value of MALAT1 in severe pneumonia, which was exhibited in Fig. 1B. The finding demonstrated the area under the curve (AUC) was 0.927, showing the possibility of MALAT1 as a satisfactory diagnostic marker. When the cut-off value was 1.5, the sensitivity (88.2%) and specificity (83.9%) indicated the superiority of MALAT1 in predicting severe CAP.

**Table 1:** Comparison of baseline demographic and laboratory data between healthy children and children with severe community-acquired pneumonia (CAP)

	Severe CAP (*n* = 93)	Healthy children (*n* = 93)	*P* value
Age, years	5.0 (4.0–7.0)	5.0 (3.0–7.0)	0.65
Male, n (%)	55 (59.1%)	58 (62.4%)	0.76
BMI, kg/m^2^	21.9 (20.0-23.8)	21.6 (19.8–23.5)	0.34
WBC (10^9^/L)	9.9 (9.0-10.8)	9.7 (8.9–10.5)	0.29
Neutrophils	15.4 (13.9–15.4)	3.5 (3.2–4.2)	< 0.01
CRP, mg/L	11.7 (12.5–13.5)	0.2 (0.1–0.2)	< 0.01
Lactate dehydrogenase, U/L	295.4 (262.7-352.9)	237.2 (212.4-259.3)	< 0.01
Lymphocyte, 10^9^/L	2.8 (1.8–3.9)	2.4 (2.2–2.6)	< 0.01
PCT, ng/mL	2.1 (1.5–2.7)	0.2 (0.1–0.2)	< 0.01

Abbreviations: CAP, community-acquired pneumonia; BMI, body mass index; WBC, white blood cell; CRP, C-reactive protein; PCT, procalcitonin

**Fig. 2:**
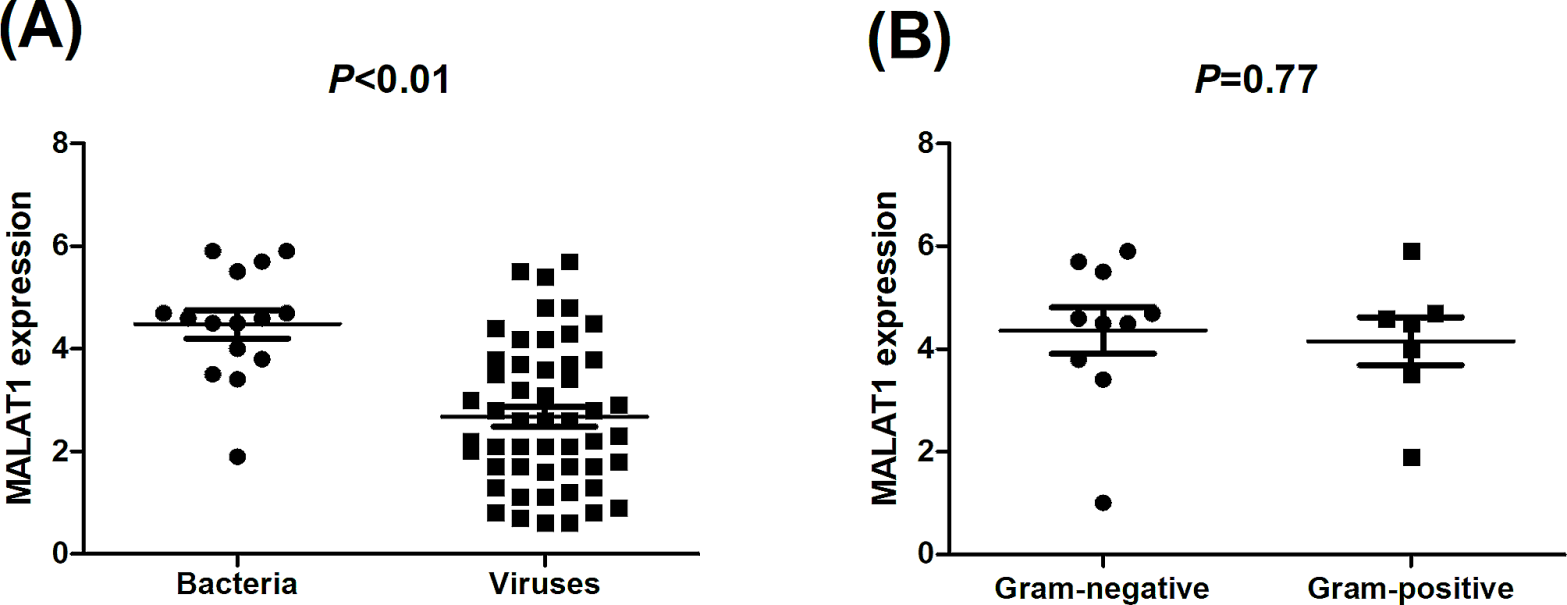
(**A**) Comparison of Metastasis-associated lung adenocarcinoma transcript 1 (MALAT1) mRNA expression levels between bacterial pneumonia and viral pneumonia; (**B**) Comparison of MALAT1 mRNA expression levels between gram-positive and gram-negative bacterial pneumonia

Table 2 summarizes the baseline demographic, laboratory, and clinical data in patients with severe CAP according to survival status. PCT level was significantly lower in survivors (*P* < 0.01). The proportions of acute lung injury/acute respiratory distress syndrome (ALI/ARDS), septic shock, heart failure, and mechanical ventilation use were significantly lower in survivals (*P* < 0.01). The differences in age, sex, BMI, WBC, neutrophils, lactate dehydrogenase, lymphocytes, CRP, PCT, pediatric intensive care unit (PICU) stay and non-invasive ventilation was not statistically significant between survivors and non-survivals (*P* > 0.05). In terms of etiology, the proportion of bacteria is significantly higher in non-survivals than in survivors (*P* < 0.05, Table 2). As shown in Fig. 2, the MALAT1 expression is significantly higher in patients detected with bacteria than those with viruses (3.8 vs. 1.7, *P* < 0.01), and the difference in MALAT1 expression between patients with gram-positive and gram-negative bacteria was not statistically significant (3.7 vs. 3.5, *P* = 0.77).

**Table 2:** Comparison of baseline demographic and laboratory data between survival and death in patients with severe community-acquired pneumonia (CAP)

	Survival (*n* = 63)	Death (*n* = 30)	*P* value
Demographics			
Age, years	6.0 (4.0–8.0)	4.5 (4.0–6.0)	0.20
Male, n (%)	39 (61.9%)	16 (53.3%)	0.50
BMI, kg/m^2^	22.0 (20.4–23.7)	21.4 (19.4–24.9)	0.47
Laboratory			
WBC (10^9^/L)	9.8 (9.0-10.8)	10.0 (9.2–10.7)	0.72
Neutrophils	15.6 (14.0-16.7)	15.1 (13.6–16.8)	0.57
CRP, mg/L	12.5 (11.6–13.5)	12.4 (12.1–13.4)	0.93
Lactate dehydrogenase, U/L	303.1 (263.8-352.6)	290.0 (259.6-353.8)	1.00
Lymphocyte, 10^9^/L	2.8 (1.7–3.9)	2.8 (2.0-4.1)	0.87
PCT, ng/mL	1.9 (1.4–2.5)	2.3 (2.0-3.3)	< 0.01
Comorbidities			
ALI/ARDS	11 (17.5%)	21 (70.0%)	< 0.01
Septic shock	4 (6.3%)	14 (46.7%)	< 0.01
Acute renal failure	5 (7.9%)	4 (13.3%)	0.46
Heart failure	1 (1.6%)	7 (23.3%)	< 0.01
PICU, days	5.0 (3.0–7.0)	5.0 (3.0–9.0)	0.55
Non-invasive ventilation	7 (11.1%)	1 (3.3%)	0.43
Mechanical ventilation	16 (25.4%)	21 (70.0%)	< 0.01
Etiology			
Viruses	37 (58.7%)	14 (46.7%)	0.50
Bacteria	4 (6.3%)	11 (36.7%)	< 0.01
Gram-positive	1 (1.6%)	6 (20.0%)	< 0.01
Gram-negative	3 (4.8%)	5 (16.7%)	0.04

Abbreviations: BMI, body mass index; WBC, white blood cell; CRP, C-reactive protein; PCT, procalcitonin; ALI/ARDS, Acute lung injury/acute respiratory distress syndrome; PICU, pediatric intensive care unit

**Fig. 3:**
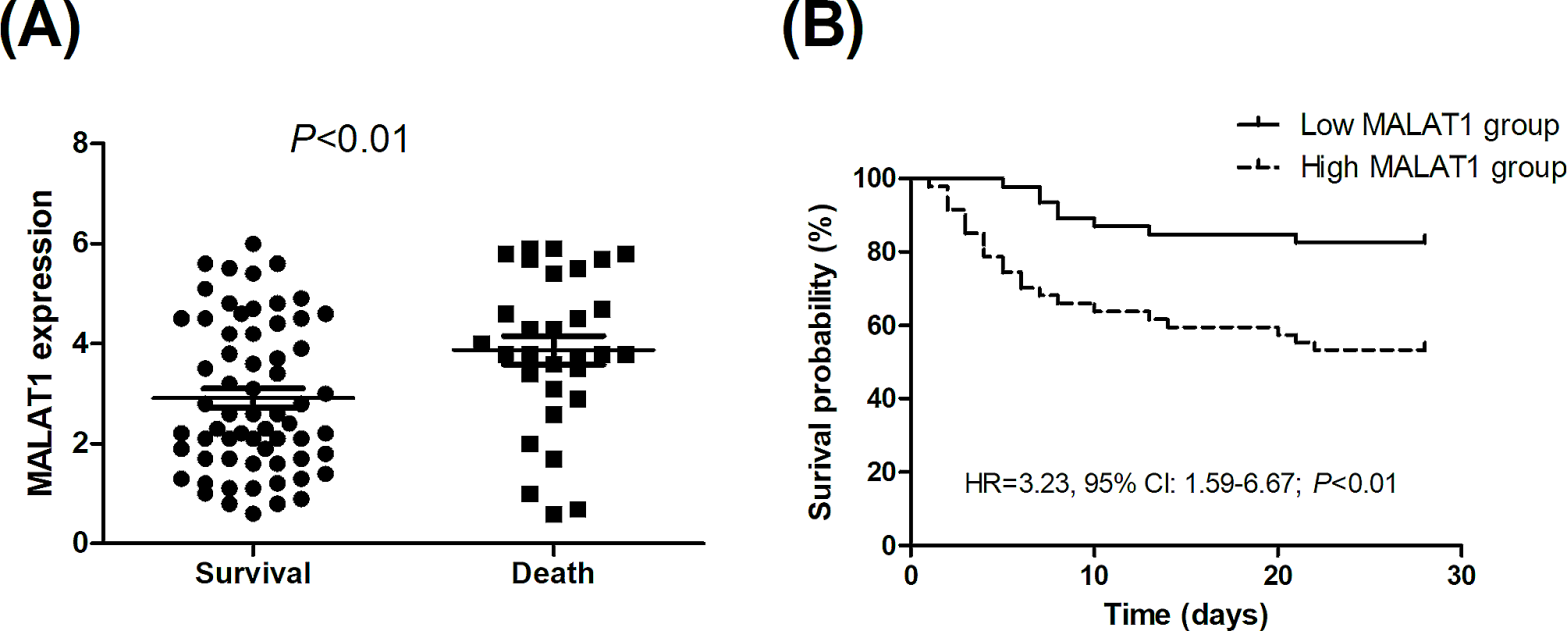
(**A**) Metastasis-associated lung adenocarcinoma transcript 1 (MALAT1) mRNA expression levels in survival and non-survival; (**B**) Kaplan-Meier survival curves in high and low-MALAT1 levels

Figure 3A illustrates that the MALAT1 expression levels were significantly higher in non-survivors compared to in survivors (3.8 vs. 2.6, *P* < 0.01). As shown in the Kaplan-Meier survival curves (Fig. 3B), subjects with higher MALAT1 levels had a higher risk of mortality than those with lower MALAT1 levels (HR = 3.23, 95% CI: 1.59–6.67; *P* < 0.01). Multivariate Cox proportional-hazards regression analysis showed that a higher MALAT1 level was significantly associated with an increased mortality risk (HR = 3.32; 95% CI: 1.05–10.47; *P* = 0.04. Table 3). The presence of ALI/ARDS (HR = 4.35; 95% CI: 1.23–15.34; *P* = 0.02) and heart failure (HR = 8.08; 95% CI: 1.85–35.39; *P* < 0.01), mechanical ventilation use (HR = 3.66; 95% CI: 1.27–10.57; *P* = 0.02), and gram-positive bacteria (HR = 14.60; 95% CI: 2.68–79.53; *P* < 0.01) were also significantly associated with increased mortality risks.

**Table 3:** Multivariate cox regression analysis for evaluating the association of MALAT1 with the risk of 28-day mortality in pediatric patients with severe community-acquired pneumonia (CAP)

	Hazard ratio	95% CI	*P* value
MALAT1 level	3.32	1.05–10.47	**0.04**
Demographics			
Age	0.97	0.77–1.23	0.82
Male	0.41	0.15–1.11	0.08
BMI	1.05	0.90–1.23	0.56
Laboratory			
WBC	0.83	0.52–1.32	0.43
Neutrophils	1.20	0.89–1.62	0.23
CRP	0.90	0.48–1.69	0.74
Lactate dehydrogenase	1.00	0.99–1.01	0.43
Lymphocyte	0.67	0.43–1.06	0.09
PCT	1.33	0.69–2.55	0.40
Comorbidities			
ALI/ARDS	4.35	1.23–15.34	**0.02**
Septic shock	1.76	0.58–5.34	0.32
Acute renal failure	4.08	0.93–17.91	0.06
Heart failure	8.08	1.85–35.39	**< 0.01**
PICU	0.91	0.76–1.10	0.33
Non-invasive ventilation	0.37	0.04–3.50	0.38
Mechanical ventilation	3.66	1.27–10.57	**0.02**
Etiology			
Viruses	3.28	0.86–12.48	0.08
Gram-positive bacteria	14.60	2.68–79.53	**< 0.01**
Gram-negative bacteria	4.08	0.53–31.62	0.18

Abbreviations: CI, confidence interval; MALAT1, metastasis-associated lung adenocarcinoma transcript 1; WBC, white blood cell; CRP, C-reactive protein; PCT, procalcitonin; ALI/ARDS, Acute lung injury/acute respiratory distress syndrome; PICU, pediatric intensive care unit

## Discussion

Severe pneumonia weakens the immune system, which finally leads to respiratory system injury, multiple organ failure, and even shock. Substantial advances have occurred in the understanding of risk factors and etiology of pediatric pneumonia, in the development of standardized case definitions, and prevention with the production of improved vaccines and treatment. Recently observations of lncRNAs in severe pneumonia provide conceivable perspectives for exploring its potential mechanism. This study is the first to evaluate the prognostic value of MALAT1 in pediatric severe CAP, indicating that pediatric patients with a higher MALAT1 level were significantly associated with a higher risk of 28-day mortality.

In this study, the increased expression of MALAT1 was determined in severe CAP patients, implying the anomalously expressed MALT1 modulated the course of CAP and might be applied as a diagnostic biomarker for discriminating severe CAP occurrence. MALAT1 can affect pneumonia by regulating vascular endothelial growth factor (VEGF), which is an important factor influencing inflammation, airway, and associated pathophysiological changes in pneumonia [4]. It also affects airway epithelial cells and regulates the NF-κB signaling pathway, which is implicated in several critical physiological functions [17]. In vivo experiments have shown that silencing of MALAT1 reduces lung inflammation caused by M. pneumoniae infection and ameliorates the injury [18].

A recent study has assessed the association of MALAT1 levels with survival in elderly patients with severe pneumonia. Yan et al. indicated that MALAT1 is highly expressed in the serum of elderly patients with severe pneumonia [13]. Furthermore, the relative expression of MALAT1 was significantly higher in the serum of patients in the death group, suggesting it may serve as a marker for the prediction of survival of these patients. In line with this previous publication, our study confirmed the predictive and prognostic value of MALAT1 in pediatric patients with severe CAP.

In addition to MALAT1, our multivariate analysis identified the other four variables (ALI/ARDS, heart failure, mechanical ventilation, and bacteria) that were independently associated with increased risk of mortality. ALI/ARDS is reported to be associated with increased pulmonary vascular permeability, decreased lung compliance, bilateral lung infiltrates, and hypoxemia. Shi et al. indicated that both the ICU mortality rate (23% versus 10%) and 28-day mortality rate (47% versus 24%) were significantly higher in the ARDS group than in the non-ARDS group [19]. Pre-existing heart failure also predicts poor outcomes following pneumonia as mortality increases by up to 50% associated with heart failure [20]. In a multicenter study by Walden et al. [21], the hazards of 28-day mortality and 6-month mortality were 2.29 (95% CI: 1.11–4.72; *P* < 0.01) and 2.68 (95% CI: 1.49–4.85; *P* < 0.01) times higher in CAP patients requiring mechanical ventilation, respectively, indicating that the need of mechanical ventilation serves as a strong predictor of a poor prognosis. Bacterial pneumonia causes more inflammation in the arteries than viral pneumonia does, which might be the reason why the MALAT1 expressions are significantly higher in patients with bacterial pneumonia than those with viral pneumonia. In addition, gram-negative bacteria usually cause mild infections, whereas gram-positive bacteria are generally more severe and more often lead to mortality [22]. Our results showed that gram-positive bacteria rather than gram-negative ones were independent prognostic factors for 28-day mortality in pediatric patients with severe CAP.

There are several limitations. Firstly, some potential confounding factors such as socioeconomic data were not measured and may cause bias to the conclusion. In addition, the role of MALAT1 on the pathological and molecular mechanisms of CAP was not elucidated in the study, requiring more in-vivo studies to verify our findings.

In conclusion, MALAT1 might be a promising marker for predicting the prognosis of severe CAP in pediatric patients.

## Data Availability

The datasets generated and/or analysed during the current study are not publicly available as it could compromise the privacy of research participants, but are available from the corresponding author on reasonable request.
